# How Many Mobile Ions Can Electrical Measurements Detect in Perovskite Solar Cells?

**DOI:** 10.1021/acsenergylett.5c00887

**Published:** 2025-04-25

**Authors:** Moritz C. Schmidt, Bruno Ehrler

**Affiliations:** LMPV-Sustainable Energy Materials Department, AMOLF, Science Park 104, 1098 XG Amsterdam, The Netherlands

In recent years, mobile ions have been assigned to various degradation mechanisms in perovskite solar cells. Some of these include nonreversible degradation, like migration into charge transport layers (CTLs)^1^ or reaction with electrodes.^2^ Others focus on the electrostatic effects due to mobile ions. Most importantly, the accumulation of a large density of mobile ions at the interface between perovskite and charge transport layers can lead to screening of the built-in potential, which can result in enhanced interface and bulk recombination, reducing the short-circuit current density and fill-factor.^3^ The accumulation of mobile ions has also been connected to a decrease in open-circuit voltage.^4^ To obtain a comprehensive understanding of the impact of mobile ions on the device physics of perovskite solar cells, accurately determining the density and diffusion coefficient of mobile ions in perovskites is of utmost importance. However, measured ion densities cover multiple orders of magnitude from 10^15^ cm^–3^ to 10^19^ cm^–3^.^3,5−7^ To determine ion densities, electrical measurements like transient current measurements, also known as bias-assisted charge extraction,^3^ capacitance frequency, also known as impedance spectroscopy,^8,9^ transient capacitance measurements, also known as transient ion drift measurements,^9^ and low-frequency Mott–Schottky measurements^5^ have been applied. Here, we illustrate that it becomes impossible to determine the ion density if it is high enough to screen a significant portion of the built-in field. To illustrate the difficulty of extracting high ion densities from the different electrical measurements, we carried out drift-diffusion simulations. For the transport layers, we chose parameters resembling thin organic transport layers 2PACz and C_60_. We assume that ionic transport is mediated by halide vacancies,^10−12^ and their charge is compensated by nonmobile negatively charged ions.^13^ We carried out the simulations for different mobile ion densities ranging from 10^16^ to 10^20^ cm^–3^, and a typical ionic conductivity σ_ion_ = *e*μ_ion_*N*_ion_ of 1.6 · 10^–10^S/cm, where *e* is the elementary charge, μ_ion_ is the ionic mobility, and *N*_ion_ is the density of mobile ions. The complete simulation parameters are listed in the Supporting Information. We emphasize that the absolute values of the presented results are only valid for the parameter set studied in this work.

The resulting simulations of the potential distribution at 0 V, steady state JV simulations, and simulations of the various techniques are shown in Figure 1. At low ion densities of 10^16^ cm^–3^, the ion density is not high enough to screen the built-in field, resulting in a significant potential drop in the perovskite, as shown in Figure 1a. Increasing the ion density leads to increased screening of the built-in field until almost no potential drops in the perovskite bulk for ion densities of 10^18^ cm^–3^ and higher. Due to the increased field screening, the bulk and surface recombination around *J*_sc_ and at low forward bias increases, resulting in a significant drop of *J*_sc_ with increasing ion densities (see Figure 1b), which has been experimentally observed.^3^

**Figure 1:**
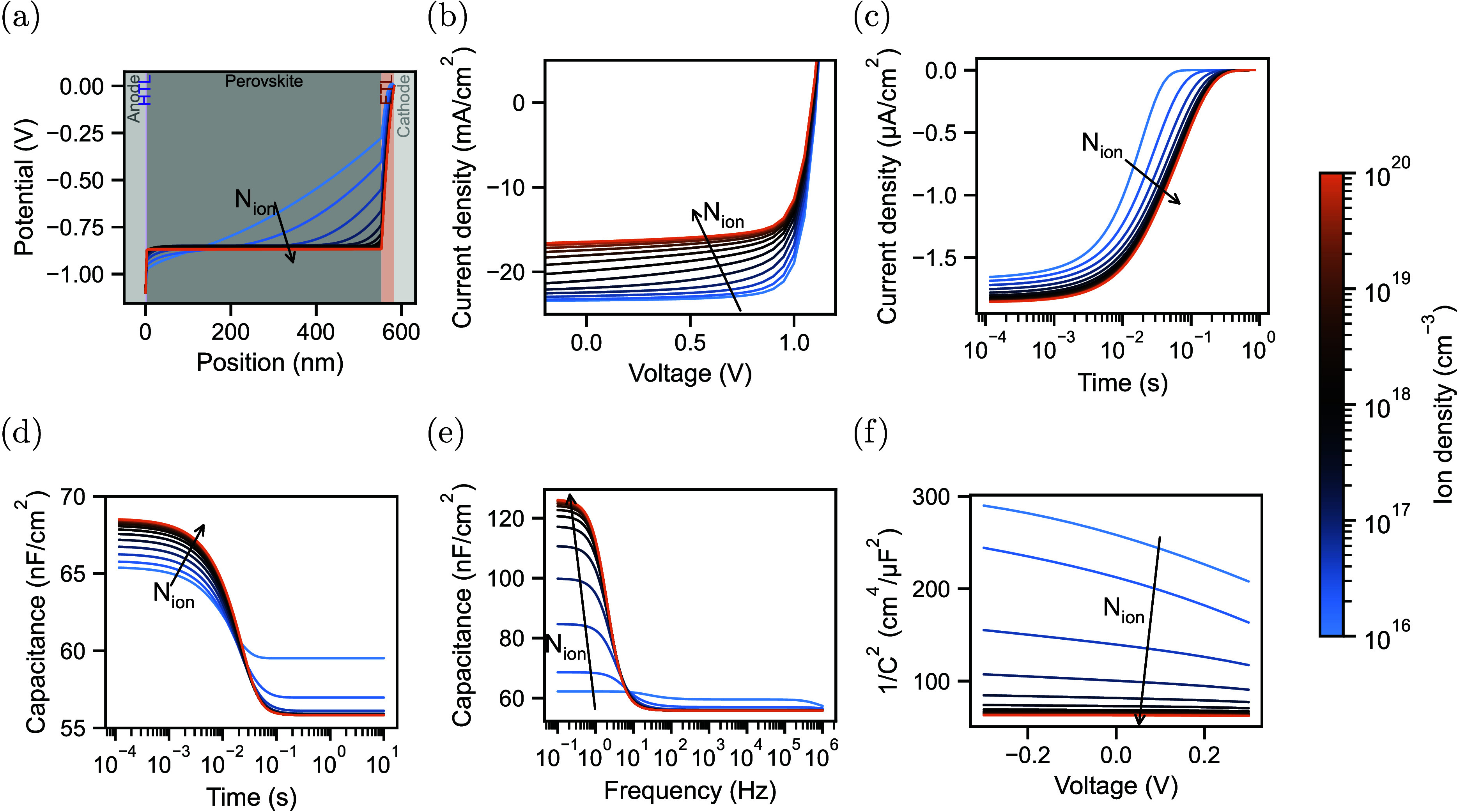
Drift diffusion simulations of a device resembling a perovskite solar cell with different ion densities. Simulation of (a) potential distribution, (b) current-density vs voltage, (c) current transient (d), capacitance transients (e), capacitance vs frequency, and (f) low-frequency Mott–Schottky.

Next, we illustrate how the screening of the built-in potential impacts the different techniques used to quantify ion densities. First, we focus on the transient measurements, transient capacitance, and transient current. In both techniques, a forward bias is applied to the device resulting in mobile ions diffusing away from the perovskite/CTL interface into the perovskite bulk.^3,14^ Then, after removing the applied bias, ions drift back to the interface, resulting in an ionic current. Additionally, the screening of the built-in potential and the change of the bulk electric field results in a displacement current. The sum of these currents is measured. Generally, the amplitude of the ionic current depends on the ionic conductivity. The integral of the current has been used to approximate the overall ion density.^3^ However, as illustrated in Figure 1c, the transients saturate for ion densities at around 10^18^ cm^–3^. With increasing ion density, more potential drops close to the interface between perovskite and CTLs, resulting in only a fraction of the ions contributing to the current. This limit for extracting high ion densities for transient current measurements has also been observed elsewhere.^5^ We note that higher ion densities than the theoretical maximum for one ion have been observed in degraded devices,^3,5^ suggesting that additional effects, like additional ions and side collection may contribute to the current transients.

In transient capacitance measurements, the modulation of the device capacitance is measured while mobile ions accumulate at the perovskite/CTL interface following a voltage pulse.^14^ This leads to a reduction of capacitance, as the accumulation of ions leads to a depletion of electronic carriers from the CTLs and consequently a reduction of the high-frequency capacitance.^14^ Both, the initial capacitance and the steady state capacitance can be impacted by mobile ions, as illustrated in Figure 1d. In the presented case, the higher ion densities lead to a larger potential drop in the perovskite layer and, consequently, a lower depletion of electronic carriers from the transport layers, resulting in a higher initial capacitance. As can be seen, at ion densities of 10^18^ cm^–3^ and higher, the initial capacitance is saturating. The steady-state capacitance depends on the level of depletion when ions accumulate at the perovskite/CTL interfaces. Here, starting at 10^17^ cm^–3^, the transport layers are depleted, leading to the same capacitance values for these ion densities. Due to the saturation of the initial capacitance, ion densities higher than 10^18^ cm^–3^ cannot be accurately determined.

Next, we focus on impedance (capacitance vs frequency) measurements. Here, the device capacitance at 0 V is measured at various frequencies. At 0 V, the mobile ions are accumulated at the perovskite/HTL interface and depleted from the perovskite/ETL interface. At high frequencies, the dielectric capacitance of the semiconductor stack, i.e., the series connection of the dielectric capacitance of the perovskite and the depletion layer capacitances of the transport layers, is probed. At low frequencies, the polarization capacitance due to mobile ions is probed, resulting in a rise, as shown in Figure 1e. The ionic conductivity determines the onset of the rise, while the amplitude depends, to some extent, on the density of ions. As shown in Figure 1e, as the density of ions increases, the low-frequency capacitance also increases until a density of around 3·10^18^ cm^–3^. At higher ion density, more ions are accumulated at the perovskite/CTL interfaces. However, the AC-potential drops in an increasingly small region close to the perovskite/CTL interface, limiting the density of excited ions and, therefore, also the capacitance.

Lastly, in low-frequency Mott–Schottky measurements, the low-frequency capacitance at small DC voltages around 0 V is measured. The DC bias modulates the depletion/accumulation layer of mobile ions at the perovskite/CTL interfaces. This modulation is then probed by determining the low-frequency capacitance.^5^Figure 1f shows the low-frequency Mott–Schottky plot for various ion densities. For low ion densities, the slope changes considerably. However, for ion densities of around 10^18^ cm^–3^, the Mott–Schottky response stabilizes, and an accurate determination of the ion density is no longer possible. Interestingly, similar to the limitation of extracting ion densities, it was previously shown that the conventional Mott–Schottky analysis also suffers from limitations when applied to perovskite solar cells to extract electronic defect densities.^15,16^

We note that the upper limit for determinable ion densities of around 10^18^ cm^–3^ is only valid for the presented device and can not be generalized. The ion density necessary to screen the built-in potential depends on numerous device parameters. These include all parameters that impact the potential of the device, specifically the potential under dark conditions. These parameters include the built-in potential, the thicknesses, doping densities, and the dielectric constants of the individual layers. For example, a smaller built-in potential would decrease the density of ions necessary to screen the built-in potential. Consequently, the maximum determinable ion density would decrease. Similarly, a larger potential drop in the CTLs, for example, due to lower dielectric constants or thicker layers, would also decrease the necessary ion density to screen the built-in potential, lowering the maximum ion density that can be determined. Parameters like the ionic diffusion coefficient or recombination velocities do not significantly impact the device’s potential in the dark. Therefore, these parameters will also not impact the maximum determinable ion density. We note that we only account for effects covered by drift-diffusion simulations. Processes like the annihilation of ionic defects^17^ can lead to a reduced field screening effect due to ionic carriers and, therefore, impact the maximum determinable ion density. Additional polarization effects at the transport layers^18^ can also impact how many ions can accumulate at the perovskite/CTL interface before the built-in potential is screened. These effects could also explain why high ion densities of up to 5·10^18^ cm^–3^ have been measured.^3^ Generally, a good approximation of the potential drop within the different layers is necessary to ensure that the extracted ion density lies in a regime where an accurate extraction is possible. For ion densities above the maximum that is possible to determine with electrical measurements, only the ionic conductivity, which depends on both ion density and mobility, can be accurately determined. The maximum determinable ion density can be increased by increasing the fraction of built-in potential that drops within the perovskite. This can, for example, be achieved by using highly doped or no transport layers. Then, significantly more potential would drop in the perovskite, leading to a higher necessary ion density to screen the built-in potential, also increasing the maximum determinable ion density.

In summary, we have shown that accurately determining ion densities becomes impossible if mobile ions screen significant parts of the built-in potential. The current transient, capacitance transient, and capacitance frequency measurements saturate at high ion densities. In low-frequency Mott–Schottky measurements, the slope saturates. Accordingly, ion densities cannot be determined accurately anymore. We additionally note that the built-in potential and the potential drops in the device can impact the maximum determinable ion density. Therefore, a good understanding and estimation of the device parameters are crucial when applying any of the studied measurement techniques to extract ion densities. To make sure that ion densities can be determined, drift-diffusion simulation can be of great help. After extracting an ion density using one of the discussed techniques, one can, for example, simulate the technique with various ion densities to determine if the regime is suitable to accurately extract ion densities.

## Supporting Information Available

The parameters for the drift-diffusion simulations (PDF)
