# Supplementary material for: How Many Mobile Ions Can Electrical Measurements Detect in Perovskite Solar Cells?
Source: ACS Energy Lett. 2025 Apr 25;10(5):2457–60. doi: 10.1021/acsenergylett.5c00887 (PMC12070455; doi:10.1021/acsenergylett.5c00887)
Supplement: Supplementary file 1 — nz5c00887_si_001.pdf [file nz5c00887_si_001.pdf]

Supporting information to: How many mobile ions can electrical measurements detect in perovskite solar cells?

Moritz C. Schmidt[†] and Bruno Ehrler[*,‡]

†*LMPV-Sustainable Energy Materials Department, AMOLF, Science Park 104, 1098 XG, Amsterdam, The Netherlands*

‡*AMOLF, Science Park 104, 1098 XG, Amsterdam, The Netherlands*

E-mail: b.ehrler@amolf.nl

**Parameter**	**Value**
Band gap perovskite (eV)	1.6
Electron affinity perovskite (eV)	3.9
Dielectric constant perovskite	62
Thickness perovskite (nm)	550
Effective density of states conduction band perovskite (cm^{-3})	$2.1 \cdot 10^{18}$
Effective density of states valence band (cm^{-3})	$2.1 \cdot 10^{18}$
Mobility electrons in perovskite (cm^2/Vs)	1
Mobility holes in perovskite (cm^2/Vs)	1
Mobile positive ion density in perovskite (cm^{-3})	variable
Electron SRH lifetime in perovskite (nm)	200
Hole SRH lifetime in perovskite (nm)	200

Electron recombination velocity at HTL/perovskite interface (cm/s)	100
Hole recombination velocity at HTL/perovskite interface (cm/s)	100
Electron recombination velocity at perovskite/ETL interface (cm/s)	1000
Hole recombination velocity at perovskite/ETL interface (cm/s)	1000
Immobile negative ion density (cm^{-3})	variable
Ionic conductivity (S/cm)	$1.6 \cdot 10^{-10}$
Band gap HTL (eV)	1.9
Electron affinity HTL (eV)	3.4
Dielectric constant HTL	3.0
Thickness HTL (nm)	3
Effective density of states conduction band HTL (cm^{-3})	$2.1 \cdot 10^{18}$
Effective density of states valence band (cm^{-3})	$2.1 \cdot 10^{18}$
Mobility holes in HTL $(\mathrm{cm}^2/\mathrm{Vs})$	$1 \cdot 10^{-4}$
Work function anode (eV)	5.2
Band gap ETL (eV)	2.0
Electron affinity ETL (eV)	4.0
Dielectric constant ETL	5.0
Thickness ETL (nm)	30
Effective density of states conduction band ETL (cm^{-3})	$2.1 \cdot 10^{18}$
Effective density of states valence band (cm^{-3})	$2.1 \cdot 10^{18}$
Mobility electrons in ETL $(\mathrm{cm}^2/\mathrm{Vs})$	$1 \cdot 10^{-4}$
Donor doping density in ETL (cm^{-3})	$3 \cdot 10^{17}$
Work function cathode (eV)	4.1
Capacitance transient probing frequency (Hz)	$20 \cdot 10^{3}$
Capacitance transient voltage pulse amplitude (V)	1.2
Current transient voltage pulse amplitude (V)	1.2

Table S1: Parameters used for the drift-diffusion simulations.
